# An Energy Efficient Routing Approach for IoT Enabled Underwater WSNs in Smart Cities

**DOI:** 10.3390/s20154116

**Published:** 2020-07-24

**Authors:** Nighat Usman, Omar Alfandi, Saeeda Usman, Asad Masood Khattak, Muhammad Awais, Bashir Hayat, Ahthasham Sajid

**Affiliations:** 1 Department of Computer Sciences, Bahria University, Lahore 54000, Pakistan; nighat.bulc@bahria.edu.pk; 2 College of Technological Innovation, Zayed University, Abu Dhabi 144534, UAE; omar.alfandi@zu.ac.ae (O.A.); asad.khattak@zu.ac.ae (A.M.K.); 3 Department of Electrical and Computer Engineering, COMSATS University Islamabad, Sahiwal 57000, Pakistan; saeeda@cuisahiwal.edu.pk; 4 School of Computing and Communications, Lancaster University, Bailrigg, Lancaster LA1 4YW, UK; m.awais11@lancaster.ac.uk; 5 Department of Computer Science, Institute of Management Sciences Peshawar, Peshawar 25000, Pakistan; 6 Department of Computer Science, Faculty of ICT, Balochistan University of Information Technology Engineering and Management Sciences Quetta, Quetta 87300, Balochistan, Pakistan; ahthasham.sajid@buitms.edu.pk

**Keywords:** underwater, wireless sensor networks, clustering, smart cities, routing protocols, IoT, acoustic signals, nodes

## Abstract

Nowadays, there is a growing trend in smart cities. Therefore, Terrestrial and Internet of Things (IoT) enabled Underwater Wireless Sensor Networks (TWSNs and IoT-UWSNs) are mostly used for observing and communicating via smart technologies. For the sake of collecting the desired information from the underwater environment, multiple acoustic sensors are deployed with limited resources, such as memory, battery, processing power, transmission range, etc. The replacement of resources for a particular node is not feasible due to the harsh underwater environment. Thus, the resources held by the node needs to be used efficiently to improve the lifetime of a network. In this paper, to support smart city vision, a terrestrial based “Away Cluster Head with Adaptive Clustering Habit” (ACH)${}^{2}$ is examined in the specified three dimensional (3-D) region inside the water. Three different cases are considered, which are: single sink at the water surface, multiple sinks at water surface,, and sinks at both water surface and inside water. “Underwater (ACH)${}^{2}$” (U-(ACH)${}^{2}$) is evaluated in each case. We have used depth in our proposed U-(ACH)${}^{2}$ to examine the performance of (ACH)${}^{2}$ in the ocean environment. Moreover, a comparative analysis is performed with state of the art routing protocols, including: Depth-based Routing (DBR) and Energy Efficient Depth-based Routing (EEDBR) protocol. Among all of the scenarios followed by case 1 and case 3, the number of packets sent and received at sink node are maximum using DEEC-(ACH)${}^{2}$ protocol. The packets drop ratio using TEEN-(ACH)${}^{2}$ protocol is less when compared to other algorithms in all scenarios. Whereas, for dead nodes DEEC-(ACH)${}^{2}$, LEACH-(ACH)${}^{2}$, and SEP-(ACH)${}^{2}$ protocols’ performance is different for every considered scenario. The simulation results shows that the proposed protocols outperform the existing ones.

## 1. Introduction

Water covers the two-third part of the earth in the form of lakes, rivers, streams, oceans, etc. These parts are explored and analyzed for underwater environmental monitoring, geosciences, hidden resource finding, military surveillance [1,2], pollution monitoring [3], etc. The aforementioned applications require attention to underwater sensing. However, the investigation by humans is not practically possible everywhere due to the harsh underwater environment.

In numerous nations around the globe, smart cities are turning into a reality. These cities add to improving residents’ satisfaction by offering types of assistance that are regularly founded on information removed from wireless sensor networks (WSN) and different components of the Internet of Things (IoT). Furthermore, policy management utilizes these smart city information to build its effectiveness by decreasing costs and offering extra services. Nonetheless, the data received at smart city servers are not prone to perturbations. Consequently, physical and PC attacks may take place. Underwater Wireless Sensor Networks gadgets are typically battery controlled and, subsequently, their lifetime is restricted. This issue prompts to lose information loss of the fundamental UWSN application. Similarly, the support cost on the Internet of Underwater Things expands with an immense number of UWSN gadgets. Furthermore, the novel attributes of UWSNs represent a few requirements, such as the high energy utilization, long engendering deferral, and node portability. To help the idea of the IoT enabled Underwater Wireless Sensor Networks (IoT-UWSNs), UWSN has developed as a promising framework. In this paper, our research focus is IoT-UWSNs applications and the challenges faced by the aforementioned. We approved the models by simulations. Therefore, researchers are focusing on moving towards the IoT-UWSNs [4,5]. IoT-UWSNs design is a novel class of IoT and is characterized as a “system of smart interconnected submerged objects”. IoT-UWSNs are required to power different practical applications, i.e., underwater investigation, condition observing and debacle avoidance. Due to the aforementioned applications, IoT-UWSN is considered to be one of the prospective advances towards smart cities.

A tree-dimensional (3-D) space, sensor network when deployed underwater monitors environmental events and takes appropriate actions according to user requirements. The objective of Underwater Wireless Sensor Networks (UWSNs) is to efficiently transmit the data packets from one place to another place using sensoring nodes [6]. There mainly exists two different forms of nodes in UWSNs: sensor nodes and sink nodes [7]. Sensor nodes are placed underwater in the specified region. Each node senses the desired input data about the neighborhood region and transmits the feedback towards the sink. However, the sink is positioned either at the water surface or inside the water and forwards these assembled data packets to the Base Station (BS).

Three different types of signals, such as radio, acoustic, and optical signals, are deployed in Wireless Sensor Networks (WSNs) [8]. Radio and optical waves cannot be used in UWSNs due to high absorption in water, and they are only suitable for small distance communication [9], while later one may suffer from high scattering [4]. However, low frequency acoustic signals can cover more distance when compared to radio waves [10]. Thus, acoustic waves are more suitable for UWSNs [11].

The nodes in UWSNs are accoutered with bounded battery capacity, according to [8,12] battery substitution is not feasible because of the harsh underwater environment. Thus, to extend network’s lifetime in a smart environment, the routing protocol should be energy efficient [13,14,15,16,17,18,19,20,21,22], as in smart cities.

Generally, three ways of routing are mostly employed in transmission for smart city communication. Firstly direct transmission; where every sensing node straightly sends a data packet to sink [23]. This may result in the depletion of nodes where the distance from the sink node is large [24,25]. The Hop by hop is the second transmission type; where each node senses data and transmits them to the sink by selecting the nearest neighbor as a forwarding node [26]. Consequently, the weight on the nodes increases, which are located closer to the sink nodes and, due to this reason, nodes die early at initial stages. Thereby, the lifetime of a network also decreases. The third one is the most interesting routing protocol to the researchers, known as clustering based routing [27]. Every sensing node underwater transmits the gathered data to its corresponding Cluster Heads (CHs) and, after that, every CH conveys its aggregated data to the BS [28]. In such a way, node to node exhaustive transmission is minimized, bandwidth consumption is reduced and network lifetime increased [29]. Therefore, the implementation of routing protocol is an emerging research area for IoT-UWSN to provision the smart city idea.

For the evaluation of our work presented here, we have considered three different node to BS scenarios. The cases are as follows. In the first scenario, single sink node is placed at the surface of the water. In the second scenario, four sink nodes are positioned at an equal distance from one another at the water surface, while, in the third scenario, two sink nodes are deployed; one node is placed at the water surface, whereas the other one is positioned inside the predefined 3-D region.

The motivation behind considering these scenarios is to discover one of the best routing protocol, which performs effectively and efficiently in a particular scenario. As we know that, in the practical world, we may face several constraints due to which we may need to deploy nodes differently. For this reason, we have considered the aforementioned scenarios and, according to these scenarios, the research work presented here shows that DEEC-(ACH)${}^{2}$ algorithm generates desired results in most of the cases, especially in terms of the number of packets sent to BS and the number of packets received at BS. TEEN-(ACH)${}^{2}$ protocols perform best for exhibiting less packet drop ratio.

The proposed scheme aims to examine the terrestrial based routing protocols’ performance in the underwater environment. In the proposed scheme, the nodes are arbitrarily located in the predetermined region, whereas sink(s) are either placed at the top of the water surface or either placed at two alternate positions. The sink one inside water while the second one at the water surface. After nodes deployment, every node newscast a HELLO packet for the sake of receiving an acknowledgment of its resources from the neighbors.

The selection of CH is a challenging task, as it directly influences the performance of the network. The most ideal node ought to be identified in order to prolong stability period of a network lifetime. The selection of CH is constrained by a number of parameters, such as the energy level of the node, the lossless transmission of data packet to the BS, and delivery of the data packet by the sensor node. Nevertheless, if CH is located in a dense area where its cluster members also exist in high range. CHs are also re-elected after each round. The energy of CH is utilized and depleted for the transmission of data packets to BS. In the case when the residual energy of a particular node does not satisfy the threshold level of a node selection of the CH, then the sensor node transmits the data packet of that node to the BS. The network stability is badly affected due to such a situation. The above listed reasons make the CH selection a challenging endeavor [30].

In the proposed study, each node evolves into a CH for a particular round if the remaining energy is exceeding the averagely computed remaining energy of the concerned network. However, in this way the selected CHs are not ideal in count, due to which different sizes of clusters are created. By having so, the load on these unbalanced clusters becomes high. Consequently, energy is consumed at a very high rate and network lifetime decreases sharply. Therefore, in our proposed scheme CHs are chosen on criteria of 10m distance and the rest of the nodes become normal nodes. An association between nodes is established, depending upon the depth to avoid backward transmission of data packets. The projected research work is an extended form of the previous terrestrial based (ACH)${}^{2}$ routing protocol [31].

The contribution of the proposed scheme is to propose an efficient design strategy for a smart city that focuses on three main approaches having the following properties. In the first scenario, we have deployed a single sink node at the surface of the water, and the whole process is executed for the first case. For the second scenario, we have deployed four sink nodes that are positioned at an equal distance from one another at the water surface, and the whole process is executed. For the third scenario, we have deployed two sink nodes, one node is placed at the water surface whereas, the other is positioned inside the predefined 3-D region, and the whole process is also executed for the third case. We have also considered Depth in every case to raise the performance of the nodes in smart underwater environment.

The rest of the research paper is arranged as: Section 2 details the literature review in which the previous papers are summarized according to their pros and cons, motivation and our proposed scheme that is U-(ACH)${}^{2}$ are discussed in Section 3, the simulation results are demonstrated and evaluated in Section 4, afterward, conclusions and future work are provided in Section 5. References are provided at the end of the paper.

## 2. Literature Review

In the last few decades to deliver reliable communication in underwater and smart environments, different routing protocols have been proposed. All of these routing protocols considered several parameters based on which they generated results in terms of network’s lifetime, dead nodes and packets received ratio at sink node. We have created two clusters based on energy that the nodes of a network hold in order to conduct our study. The protocols that consider only homogeneous energy are grouped into one cluster while the protocols that only consider heterogeneous energy are grouped into the other cluster. The protocols under cluster 1 and cluster 2 are discussed below:

### 2.1. Cluster-1 Protocols Holding Homogeneous Energy

In [32], Depth-based Routing (DBR), a depth based proactive routing protocol, is proposed, where multiple data packets are forwarded hop by hop, depending upon depth difference. DBR shows that its stability period is very poor, as low-depth nodes die in the very initial stages. Because of multi-hop, energy consumption while data transmission increases and, thus, the lifetime if a network decreases [33,34,35].

Extended forms of DBR are EEDBR [36,37], and CDBR [38] were proposed, where the network’s lifespan is increased by limiting the number of forwarding nodes. These nodes are nominated, depending upon the depth as well as residual energy, which prolongs the lifetime of nodes. However, in the dense network, these protocols do not perform well as the load on medium depth nodes increases. Hence, these kinds of nodes die sharply at initial stages.

A homogeneous proactive routing protocol namely CoDBR is proposed in [39], in which relay nodes are nominated, depending upon the depth. The relay nodes that are selected forwards the data packets cooperatively to the sink node. By considering cooperative routing at the network layer, the throughput and reliability of the network increases. However, the redundant transmission of similar data packet occurs due to which high energy is consumed. Like DBR, it also neglects residual energy and link condition and, besides these shortcomings at a very initial level, nodes having low depth die because of fixed depth threshold $d_{th}$ [40].

In [41], the authors proposed a Reliable and Energy efficient Pressure-Based Routing (RE-PBR) protocol for UWSNs. RE-PBR considers three factors, including join quality, depth, and remaining energy for adjusting energy utilization and solid information conveyance. In particular, the quality is assessed by utilizing the triangle metric technique. A lightweight data obtaining algorithm is created for productive information revelation of the system. Multi-metric data sending calculation is planned dependent on course cost estimation, which uses leftover energy and connection quality. Simulations were performed in NS-2 with Aqua-Sim to assess the exhibition of RE-PBR. The proposed protocol is equated with the DBR and EEDBR. According to the authors, RE-PBR performs better in the case of end to end delay, energy utilization, and network lifetime range. However, the limitation of this technique is the consumption of long time duration.

The authors of improved Adaptive Mobility of Courier nodes in Threshold-optimized DBR (iAMCTD) [42,43], proposed a Forwarding-Function ($F_{F}$) based routing protocol for time-sensitive applications in which not only depth and residual energy are considered. Moreover, it deals with the Signal to Noise Ratio (SNR) as well. To reduce the transmission loss, an optimal Holding Time ($H_{r}$) is calculated that ultimately enhances network lifetime. The depth threshold varies following the density of a network, which requires network information on a regular interval basis. Due to this variation, a list of eligible neighbors increases and causes a reduction in critical data deficits and end to end delay.

Mobile sinks are considered in a cooperative based routing protocol, named as Depth and Energy Aware Dominating Set (DEADS) [44]. The protocol considers three stages while performing. During the first stage, neighbors are selected. Domain Set (DS) and Cooperative Courier (CC) are selected from neighbors while the second stage. Afterwards, data are sensed and forwarded to the sink depending upon the threshold value in the data forwarding phase. Improved throughput and decline in packet drop value is gained in exchange of high energy depletion.

In Adaptive Cooperation in EEDBR for UWSN (ACE) [45,46], single and double re-transmission the procedure is utilized in order to expand the throughput. The maximum number of nodes joined in retransmission progression results in maximum sets of packets collected at the sink. In the ACE protocol, the Bit Error Rate (BER) is more than 50%. A packet is dropped when it has no neighbor to send information towards the sink. By having single and double retransmission in ACE results in less packet drop.

LEACH [47,48], is a primary known clustering based protocol. The primary goal of LEACH is to introduce local cluster based communication by reducing the global transformation of data packets. A threshold value is considered and an arbitrary number is generated by all the nodes upon which selection of CHs depends. In the case that the created arbitrary number is less when comparing it to the value of the considered threshold, at that moment the particular node is nominated as CH or vise versa. However, in this way, CHs are randomly selected due to which variation in size of clusters is introduced. These variations directly affect the node(s) energy level harshly and, thus, network lifetime decreases.

The author’s goal in the proposed protocol TEEN [49] is to detect the environment and react at the exact instant, when the desired information is sensed. It also selects CHs and checks for threshold value as well as the status of previous rounds, like LEACH. However, during the transformation of data packets, unlike LEACH, a condition is required to be satisfied in which hard and soft thresholds are defined. The sensor node forwards for the first time when the sensed value is total up to the hard threshold value. However, in a case to transmit for the second time, the sensed value should be larger than the hard threshold and should be either larger or equal to the soft threshold value. Like LEACH, energy is vigorously depleted here and, as a result, network throughput decreases sharply.

### 2.2. Cluster-2 Protocols Holding Heterogeneous Energy

In [50], depth and noise-aware routing (DNAR) and cooperative DNAR (Co-DNAR) are proposed. DNAR fuses the degree of link clamor in grouping with the depth of a node to choose the subsequent data sending node. However, the data transmitted over a solitary link may get affected by the unpredictable behavior of the channel. Consequently, a cooperative scheme is added to the called DNAR, which uses source-relay-destination triplets. Co-DNAR diminishes the likelihood of data defilement, for the transmission over a solitary source-destination interface. The authors argue that network stability is increased in Co-DNAR; however, more time is consumed to choose the transfer relay nodes and, thus, Co-DNAR produces the maximum delay.

The structure of two directing calculations EERD and CoEERD for U-WSNs is proposed in [51]. The EERD limits energy utilization, while the CoEERD improves the reliability of a network. The two plans use information sender nodes dependent on the weighting capacity factors (least Bit Error Rate, highest residual energy, and smallest distance). According to the author, depending upon the aforementioned and utilizing a single route, the EERD efficiently uses the energy of a network and it also extends the stability period of a network. However, the proposed EERD protocol is unreliable and inefficient under the unpredictable behavior of water by choosing a single way. The CoEERD protocol is proposed for achieving the reliability of the network. The concept of multi-way routing is introduced here. A similar standard is characterized for the information forwarder nodes choice and collaboration is held among the relay nodes and destination nodes. The authors argue that energy is efficiently utilized and the packets are reliably transferred to the sink node. However, likewise, Co-DNAR, more time is consumed to choose the transfer relay nodes and, thus, Co-EERD also produces the maximum delay.

Authors proposed a protocol for heterogeneous networks, where all of the nodes in a network are supplied with two distinct levels of energy SEP [52]. Depending upon the energy, nodes are divided into two classes that are advanced and normal nodes. α times more energy is provided to the advanced nodes concerning the normal nodes. Due to this varying energy distribution, advanced nodes have a higher possibility to evolve into CH. In any situation, the sensor node first forwards the packet to CH and then this respective CH is responsible for the next step, even if the sensor node is at a very nearest position to the BS than a CH. This causes extra consumption of energy and the energy diminution of nodes. Therefore, the lifetime of a network shrinks.

A heterogeneous routing protocol DEEC [53], in which nodes have different initial energies. Node(s) having higher energy than the averagely computed energy of the network becomes CH(s), these CHs will transmit the aggregated packets to the sink. Because of varying energy distribution, high energy nodes are selected as CHs repeatedly. Hence, the network lifetime increases. CH selection criteria are the same as in LEACH; however, here the possibility of the node to evolve into CH is calculated with a distinct equation, which is explained in (ACH)${}^{2}$ [54]. In (ACH)${}^{2}$, the author highlighted the major issues, like unbalanced cluster size, backward forwarding, as in LEACH, TEEN, SEP, and DEEC. To thoroughly understand the existing protocols, a comparison chart of these existing schemes is shown in Table 1.

The research works that have been presented here have been conducted on underwater nodes to collect data where optimum performance is obtained. Throughput maximization and improved lifetime of a network are the desired parameters in the proposed study.

## 3. Motivation for U-${\left(\mathrm{ACH}\right)}^{2}$: Underwater Implementation of ${\left(\mathrm{ACH}\right)}^{2}$

New challenges are observed to deploy a clustering based underwater acoustic sensor network in a very large area. Limitations of the underwater environment are: sensor nodes in underwater are having limited energy, low bandwidth, and small communication range [55]. To overcome these deficiencies, reliable transmission of data packet and survivability of the network for a long duration is required for smart cities.

**Figure 1:**
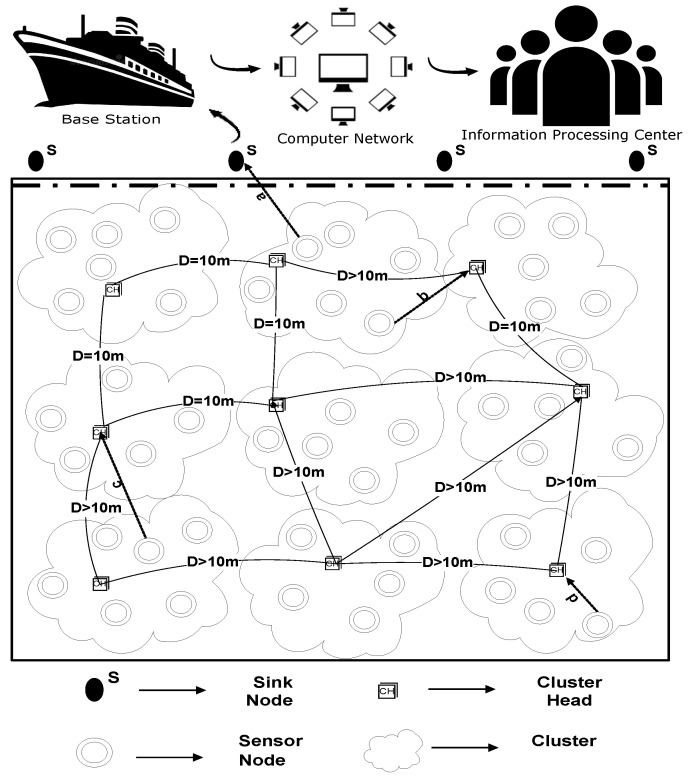
General Architecture of U-(ACH)${}^{2}$.

Many energy efficient underwater routing protocols are proposed to date; nevertheless, the desired parameters are still yet to be achieved. Challenges that are faced by underwater routing protocols are low throughput, latency in data transmission, and loss of energy at higher levels of speed. The demands of smart cities are demanding in terms of parallel processing and the shortcomings listed above need to be addressed in the existing routing protocols. In order to overcome the shortcomings, to meet the modern research criteria of a smart city, a routing protocol, namely: U-(ACH)${}^{2}$, is proposed (general architecture is shown in Figure 1). To examine the efficiency of the projected routing protocol, a terrestrial-based (ACH)${}^{2}$ routing protocol is implemented in the IoT enabled underwater environment and the effect on throughput and energy consumption is examined by using an extra feature, i.e., “depth” in it.

A terrestrial based routing algorithm (ACH)${}^{2}$ is modified in this study, in which the challenges of the underwater environment are examined. To accomplish the objectives, such as: reduced energy consumption and maximization of the throughput of sensor nodes, a new scheme in underwater is proposed, named U-(ACH)${}^{2}$. In the proposed scheme, both homogeneous and heterogeneous acoustic modems are used to get the optimum result.

### Network Model

We have considered an underwater wireless network, where we dealt with three types of nodes. Normal nodes gather information regarding the surrounding region and transmit it to the CH node. CH will further send the aggregated packets to the sink node. Normal nodes can also forward the sensed data straight to the sink if they lie nearer to the sink node. In our proposed scheme, the sink is either positioned at the top of the water surface or specified otherwise.

One of the most famous techniques used for the sharing of location information is localization. In order to start network configuration, every node in WSN requires the area IDs, link status, relative coordinates, and received signal strengths of all other nodes in order to transmit data packets. However, this technique is itself a big technical challenge for researchers [56]. U-(ACH)${}^{2}$ is a localization based routing protocol, where all sensor nodes newscast a HELLO packet to the entire structure. In this study, we have used a range-free localization algorithm. In this technique, the mobile beacon node with an antenna is utilized to provide the data regarding distance and location for every other anonymous node. By doing so, all of the nodes get localized by their neighbors. However, the node with less remaining energy than the required energy cannot send a HELLO packet, since these nodes are not able to participate in further transmission. At the point when a node gets a HELLO packet from its neighbor, it examines the remaining energy and signal strength of the neighbor under certain conditions. If the neighbor meets the given conditions of residual energy and signal strength, then the neighbor turns into the next hop for transmitting the data to the next level. Depending upon the information provided in HELLO packets, every node attains complete information of all other sensor nodes and sink node(s).

In the proposed study, “switch to next node” is a function that is considered and called when a current node is dead or at the time of traversing for re-election, the node generates no response. The reason behind for calling switching function is that the node contains very less residual energy than the required energy and it cannot broadcast the HELLO packet. Furthermore, the effected node cannot participate anymore in the transmission of data packets and, hence, becomes isolated.

**Figure 2:**
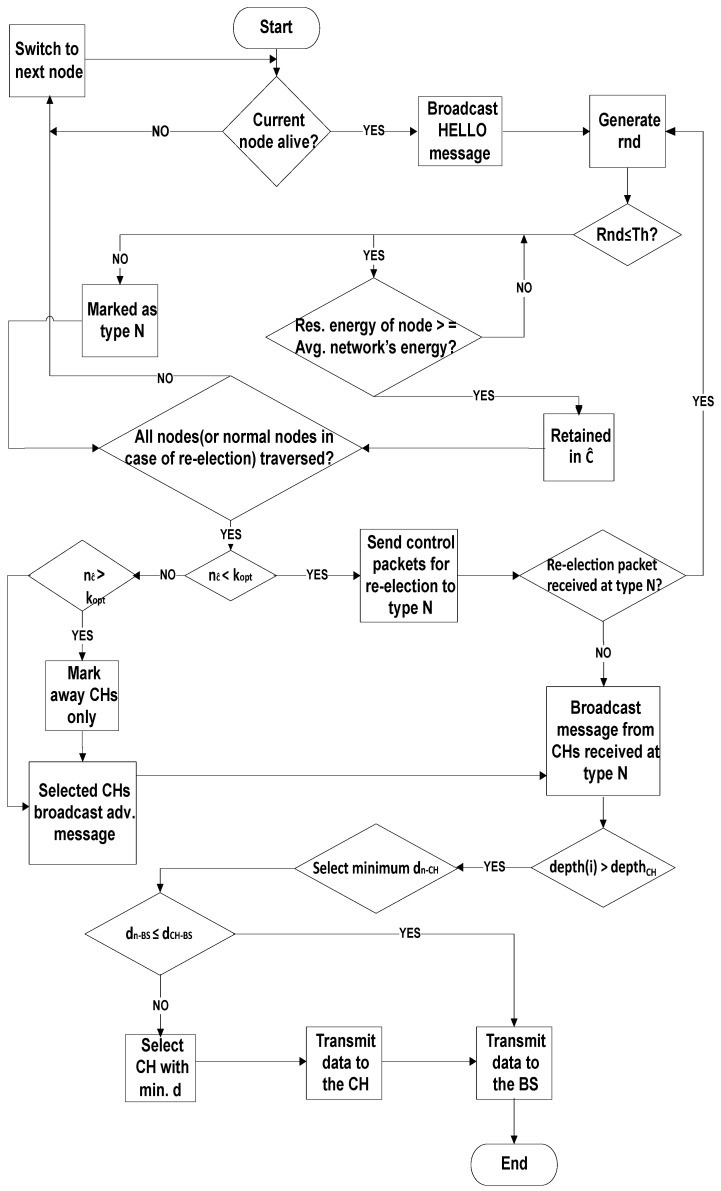
Flowchart of U-(ACH)${}^{2}$.

U-(ACH)${}^{2}$ is a clustering based routing protocol, where CHs are selected on the basis of remaining energy. The remaining energy needs to be examined in order to select CHs. Therefore, the nodes having superlative remaining power are preferred [57]. For the sake of reducing the load on CHs, the optimum number of clusters is selected that results in balanced cluster size. For this, a selection criterion is defined that depends upon the 10 m distance of a selected CH from other elected CH, as demonstrated in Figure 2. The reasoning behind the distance measure is to balance the load factor on each cluster. By doing so, many clusters would be equally loaded with the associated cluster members otherwise some clusters would have many associated cluster members while other clusters may be found under load. To avoid this controversy, the distance measure is considered in this study.

As per the results detailed in [58], the ideal number of CHs for a network containing 100-nodes is proposed to be nearby 3–5. In any case, it ought to be noticed that the rate proposed by [23] is determined dependent on the direct correspondence of CHs with the BS and, consequently, for the structures that utilize multi-hop transmission to move the collected data of CHs to the sink node or the schemes with various energy utilization, the forms may differ.

In the proposed study, in total 225 nodes were selected. According to [59] and [60], the range of distance is estimated in the range of 9 < Kopt < 11 when the base station is placed away from the field. Therefore, we have also considered, at most, 10 clusters at a time and, based on the Euclidean Distance measure, the CHs are selected. On average, 10m distance is considered for the selection of CHs to communicate in the desired network.

Each sensor node will sense the environment, collect the information of interest, and will forward its data packet to its nearest low depth CH. After this process, the selected CHs transmit the collective data straight towards the sink node(s). Contrarily, if the computed span in sensor node and sink is minimum than the CH, and node lies above the minimum distance CH, then the node will directly send data to sink.

In order to conduct the simulation, for each scenario, we have considered 1000 rounds and for each round, we have assumed the number of nodes ‘N’ as 225. We have computed the number of nodes that were participating in sending the 10 kb data to BS in each round from the list of 225 nodes. Each carrier node transmits the 10 kb data packet to the BS. At the base station, the attainment of 10 kb data packet is equal to the one data packet. Likewise, we divide the received kb data to the 10 kb which shows the received data packets.

Secondly, the number of packets a node sends depends upon the residual energy a sensor node holds and a position where it is located. If a sensor node is located near to the CH or sink node then ultimately packet drop ratio will be less and more packets will be sent by a node to the BS. In an alternative case less number of packets, a node will transmit if its residual energy is less and it is located far away from the CH or sink node.

WSNs are divided into two classes while considering energy, homogeneous, and heterogeneous [52]. We have considered four routing protocols in which two of them are homogenous, such as (LEACH, TEEN) and the rest of the two are heterogenous, which are (SEP, DEEC). The working principle of LEACH-(ACH)${}^{2}$, TEEN-(ACH)${}^{2}$, SEP-(ACH)${}^{2}$, CH election, selection, and DEEC-(ACH)${}^{2}$ is demonstrated in Algorithms 1–5, respectively.
**Algorithm 1** LEACH-ACH${}^{2}$.
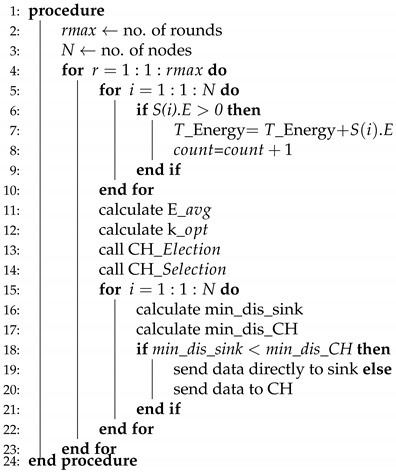


LEACH-ACH${}^{2}$, as shown in Algorithm 1, is the simplest algorithm where no hard/soft threshold and advance energy is required. Initially the number of nodes and rounds are specified for simulation. Next, the energy of node is computed to identify whether it is alive or dead. After wards, total energy of the node is computed. The count is incremented from node 1 to node 2 and so on until no dead node is found.

Afterwards, the E_avg and K_opt values are computed. A call is generated to the CH-Election function and then CH-selection function. The distance from sensor node to CH and sink node is computed. Sensor node will transmit data to the CH if its distance value is less when compared to sink node or vice versa.

TEEN-ACH${}^{2}$, as shown in Algorithm 2, is a two-level clustering scheme, where the CH broadcasts two thresholds to its associated members. The first is a hard threshold while the other is known as a soft threshold, which demonstrated the variation in the value of a sensed feature. The hard threshold is responsible for sending data only when the sensed feature range is satisfied.

Initially, the number of nodes and rounds is specified for simulation. During the transformation of data packets, a condition is required to be satisfied in which hard and soft thresholds are defined. Afterwards, the energy of the node is computed to identify whether it is alive or dead. The next step is the computation of total energy of the node. The count is incremented from node 1 to node 2, and so on, until no dead node is found.
**Algorithm 2** TEEN-ACH${}^{2}$.
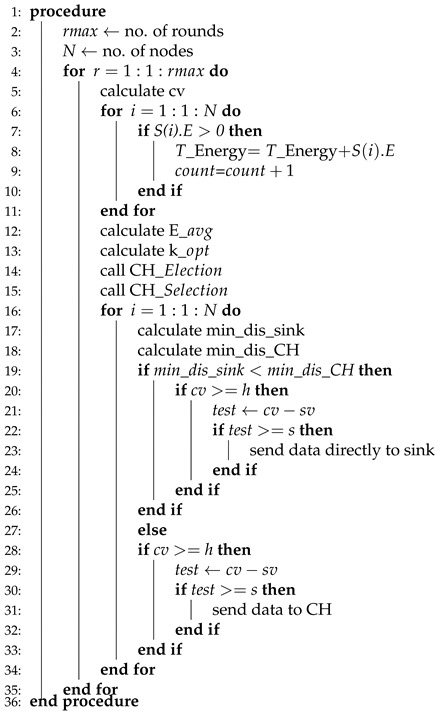


Next, the E_avg and K_opt values are computed. A call is generated to the CH-Election function and then CH-selection function. The distance from the sensor node to CH and sink node is computed. The sensor node will transmit data to the CH if its distance value is less when compared to the sink node or vice versa. The sensor node forwards for the first time when the sensed value is total up to the hard threshold value. However, in a case to transmit for the second time, the sensed value should be larger than the hard threshold and it should be either larger or equal to the soft threshold value as shown in the algorithm.
**Algorithm 3** SEP-ACH${}^{2}$.
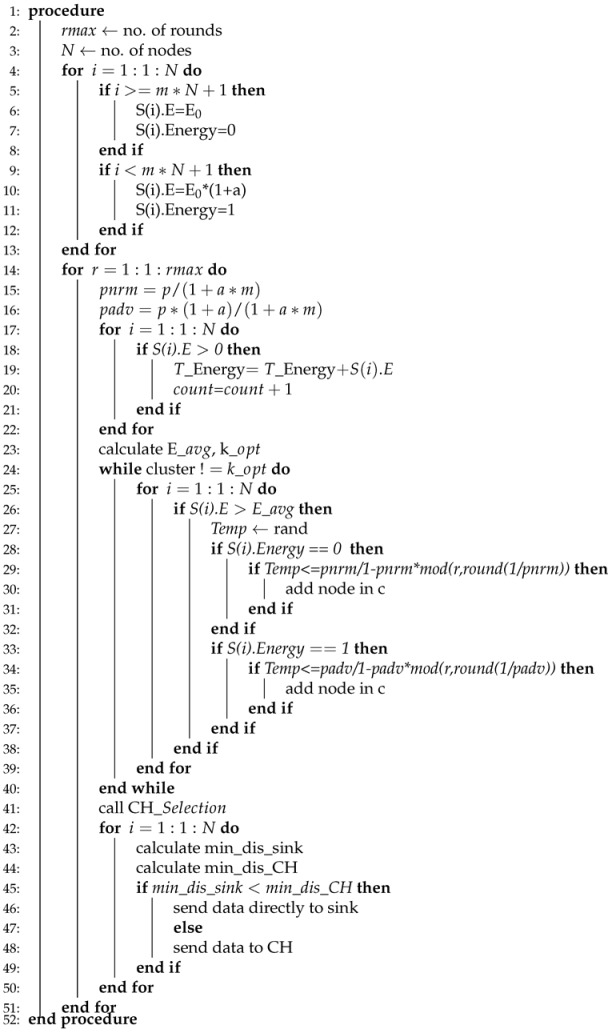


Like DEEC-ACH${}^{2}$, the SEP-ACH${}^{2}$ scheme is also based on heterogeneous scheme comprising of two different levels of energies, as shown in Algorithm 3. Initially, the number of nodes and rounds is specified for simulation, m is the percentage of advance nodes. E0 denotes the normal node, while E0⁢(1+a) denotes the advance nodes. Here, α time more energy is assigned to the advance nodes.

The probability of normal and advance nodes are computed. After the computation of probabilities, the energy of the node is computed to identify whether it is alive or dead. Afterwards, the total energy of the node is computed. The count is incremented from node 1 to node 2 and so on until no dead node is found. Afterwards, the E_avg and K_opt values are computed. Like other schemes here, CH-election and selection will not be called, because, in SEP-ACH${}^{2}$, only CH is responsible for transmitting the data. If the sensor node lies near to the sink node, still normal node is not allowed to send data directly to the sink node. Due to this nature of SEP-ACH${}^{2}$ scheme, the network’s lifetime is sharply decreased at a very high speed.
**Algorithm 4** CH election, selection.
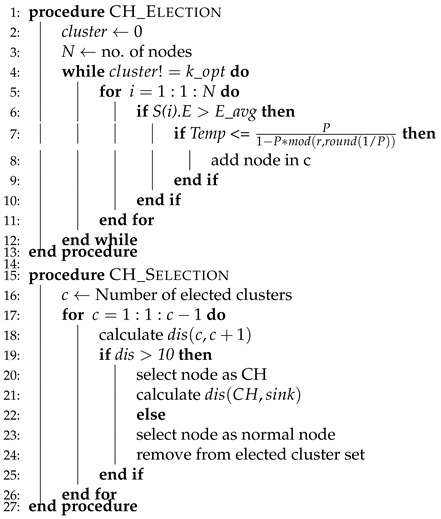


For CH-election, initially cluster variable is assigned 0 value and the number of nodes is considered in a way, as already considered. For a node to become CH, its residual energy needs to be more than the average residual energy of the network, if the condition satisfies, then the sensor node is elected to be a CH. The CHs are elected until the K_opt value is reached, as shown in Algorithm 4.

For selection, the distance from all of the elected nodes towards every next node is computed, if the distance is greater than the specified value i.e., 10m then the elected node is selected as CH or in alternative case elected node again becomes a normal sensor node. For each round, election and selection process executes.
**Algorithm 5** DEEC-ACH${}^{2}$.
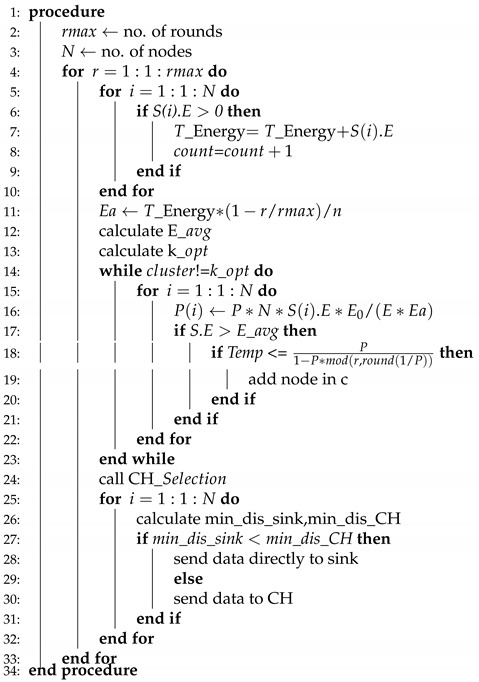


DEEC-ACH${}^{2}$ is a Heterogeneous WSN that comprises of two or various sorts of sensor nodes having different energy levels as shown in Algorithm 5. Initially, the number of nodes and rounds is specified for simulation. Next, the energy of the node is computed to identify whether it is alive or dead. Afterwards, the total energy of a node is computed. The count is incremented from node 1 to node 2 and so on until no dead node is found.

The DEEC-ACH${}^{2}$ convention depends on a two-level heterogeneous WSN in which the sensor nodes are equipped with normal E0 and advance Ea energy levels. The normal energy of the network is given as

(1) Ea=T_Energy∗(1−r/rmax/n)

After computing E_avg, we need to compute K_opt in order to create the number of desired clusters. Toward the start of each round, the choice with regards to whether the nodes are CH is chosen by the limit bound. The limit bound is suggested, as below. It is imperative to take note of that ideal likelihood (P(i)) is somewhere in the range of 0 and 1, which is the part staying in the opposite of the P(i) with r. This is the reason mod is utilized. This leftover is deducted by 1 and Temp is determined.

if S.E > E_avg then

(2) $$\frac{P}{1-P*mod(r,round(1/P))}$$

otherwise zero.

Next, CH-selection code is called to compute the distance. For a node, to select CH, if the distance from the sink node is less when compared to the distance from the cluster, then a node will directly send its data packet to the sink node. This is how DEEC-ACH${}^{2}$ works.

In subject to the system’s performance, the list of metrics for evaluation purposes are considered, which are as follows:

The first metric is the Stability period, which is the timestamp since the creation of network till all the nodes alive. Next is the Un-stability period which is the timestamp from the end of stability period till the end of the network. Afterwards, the number of packets send to BS is the metric that shows the total number of packets from the start of the network that are straightly sent to the base station. The number of packets dropped occurs due to attenuation noise and link status packets dropped. The next metric is Throughput, which demonstrates the number of data packets successfully collected at the Base Station per round. The last metric that we considered to evaluate the scheme is the computation of the number of Dead nodes having zero energy.

To gain the ideal results, three different ways for both homogeneous and heterogeneous routing protocols are projected and discussed, which are as follows: positioning single sink at water surface; positioning multiple sinks at water surface; and locating sinks at both positions, water surface and underwater.

## 4. Results and Discussion

According to the conducted proposed study, the results are generated based on some predefined performance measures are as follows:

### 4.1. Performance Evaluation

In the proposed study we have performed the comparative analysis with the benchmarks, which are DBR and EEDBR. To measure the performance of the proposed study, the behavior of underwater (ACH)${}^{2}$ is equated with the DBR and EEDBR protocols. The benchmarks are still considered and utilized by the organizations to gather data from the environment. Furthermore, we have also used the extended form of LEACH, DEEC, TEEN, and SEP routing protocols to analyze the performance of each protocol underwater. All of the listed routing protocols perform according to the considered scenarios. By doing so, we have obtained the promising results, which will assist the researchers in further exploration of the routing protocols.

For simulation, we have used MATLAB (Mathworks Inc., USA) to measure the performance of routing protocols. The list of parameters with their values are considered, as shown in Table 2 below:

For all of the aforementioned scenarios, as discussed in Section 1, we believe that it is mandatory to claim here that it is not possible to reproduce the same results for the next time because the nodes are deployed randomly underwater and, due to unpredictable nature of water nodes, can get scattered.

#### 4.1.1. U-(ACH)${}^{2}$ with One Sink at Water Surface

**Figure 3:**
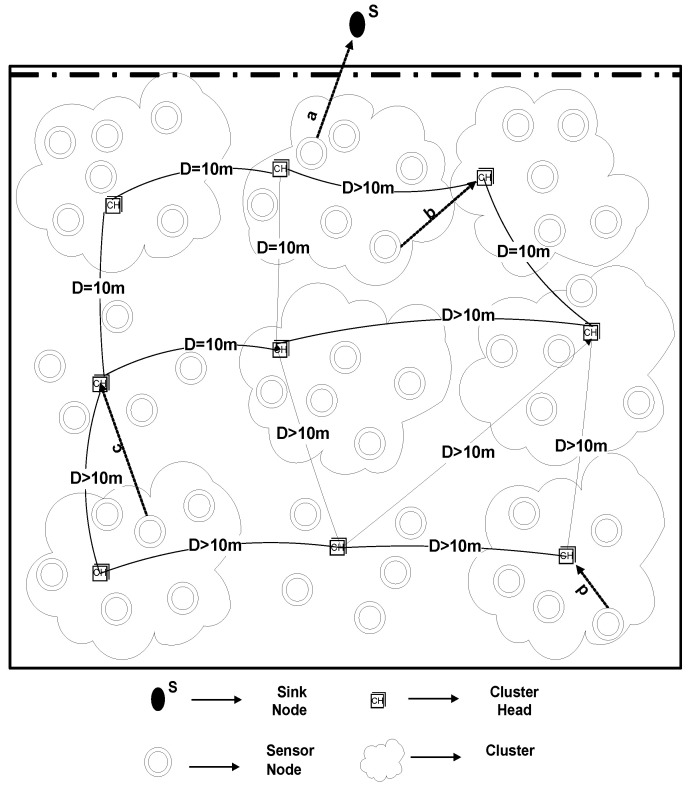
U-(ACH)${}^{2}$ with One Sink at Water Surface.

**Figure 4:**
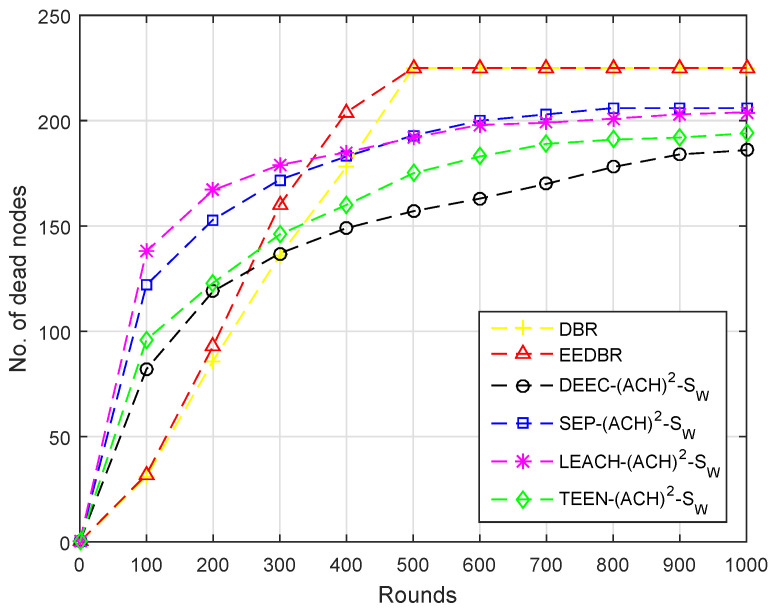
Number of Dead Nodes.

To conduct the simulation, for each scenario, we have considered 1000 rounds and, for each round, we have assumed the number of nodes ‘N’ as 225, as shown in the proposed study. We have considered 1000 rounds and for each round, these nodes perform their desired activity. The results are obtained from several runs in order to analyze the diversity among results. For each simulation, a very minute variation was found in the results which were approximately equal to the previous simulation results. The reason behind the variation is the random deployment of sensor nodes. On average, the results were the same for each run. The simulation results for the U-(ACH)${}^{2}$ with one sink at water surface scenario are shown in Figure 3 and discussed below:

Figure 4 shows the comparison of dead nodes in various routing protocols. In DBR only depth considered so at an earlier stage low depth nodes die because of high data forwarding rate. However, in other routing protocols initially load on the nodes having high residual energy is high, thus resulting in the sharp depletion of nodes. Secondly, there is only one sink in the network, which is deployed at the top, so both nodes and CHs have to transmit data packets towards the top, which requires more energy.

**Figure 5:**
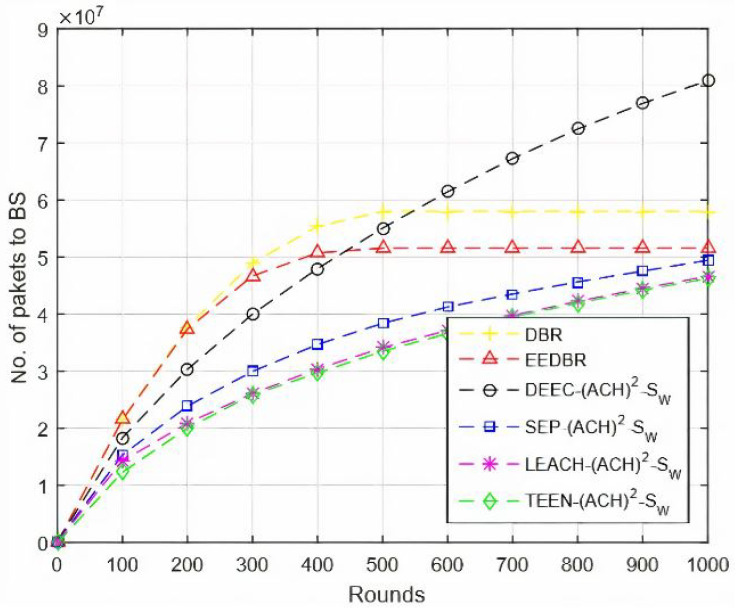
Number of Packets Sent to Base Station (BS).

In Figure 5, DEEC-(ACH)${}^{2}$ performs well when comparing with other routing protocols. As DEEC-(ACH)${}^{2}$ is a multilevel heterogeneous routing protocol because of which nodes kept alive for longer duration and, thus, large number of packets are disseminated to the sink. In DBR and EEDBR, each node sends packets through various tracks to the sink that is why results in the maximum number of packets sent. In LEACH-(ACH)${}^{2}$, all nodes have the same initial energy; hence, nodes will die soon and fewer packets will arrive at the sink. In TEEN-(ACH)${}^{2}$, packets are transmitted on the basis of the soft and hard threshold, due to which less number of packets will hit the BS.

**Figure 6:**
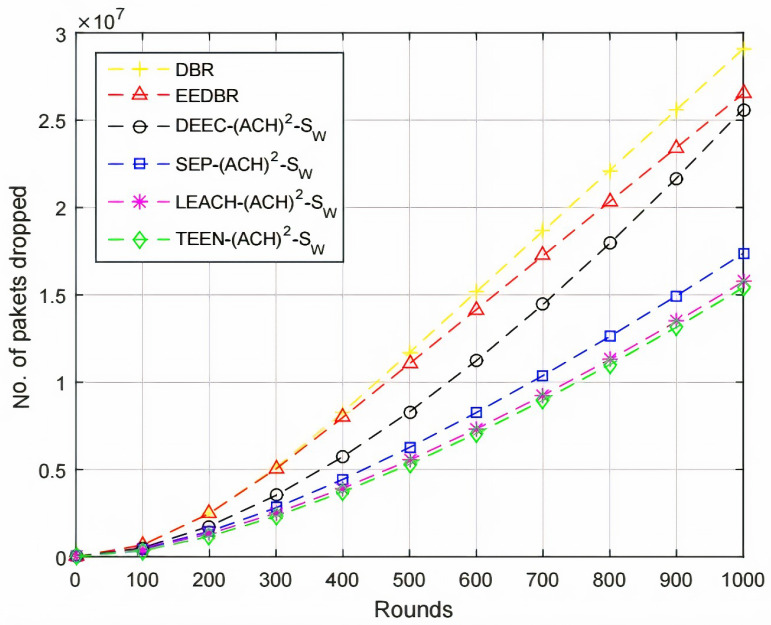
Number of Packets Dropped.

Several packets dropped are in direct relation with the number of packets sent to sink, Figure 6, shows that the number of packets dropped is high in DEEC-(ACH)${}^{2}$, because DEEC-(ACH)${}^{2}$ sent maximum number of packets. High residual energy is consumed in transmitting data to the sink. DEEC-(ACH)${}^{2}$ having higher energy will send more packets successfully to sink than others. In DBR and EEDBR, packet is forwarded through multiple directions, so the packet received first is considered as received and the rest are dropped.

**Figure 7:**
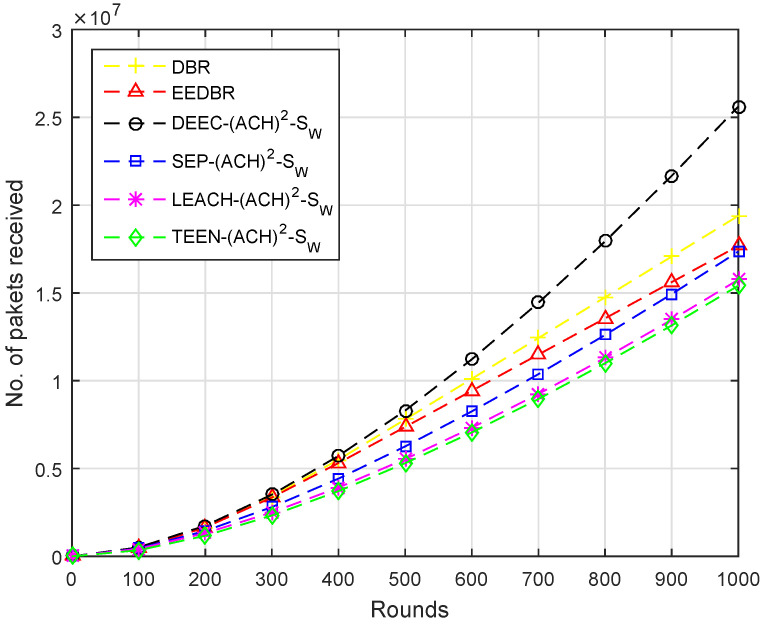
Number of Packets Received at BS.

In Figure 7, maximum packets are received by DEEC-(ACH)${}^{2}$ as it sends a large set of packets towards the sink. DBR sends packets to sink in multi-hop fashion, hence more packets received at BS. The rest of the protocols show similar behavior in this scenario.

The performance of proposed schemes is demonstrated in Table 3 for scenario-I. It can be seen that the DEEC-(ACH)${}^{2}$ routing protocol outperforms here.

#### 4.1.2. U-(ACH)${}^{2}$ with Four Sinks at Water Surface

**Figure 8:**
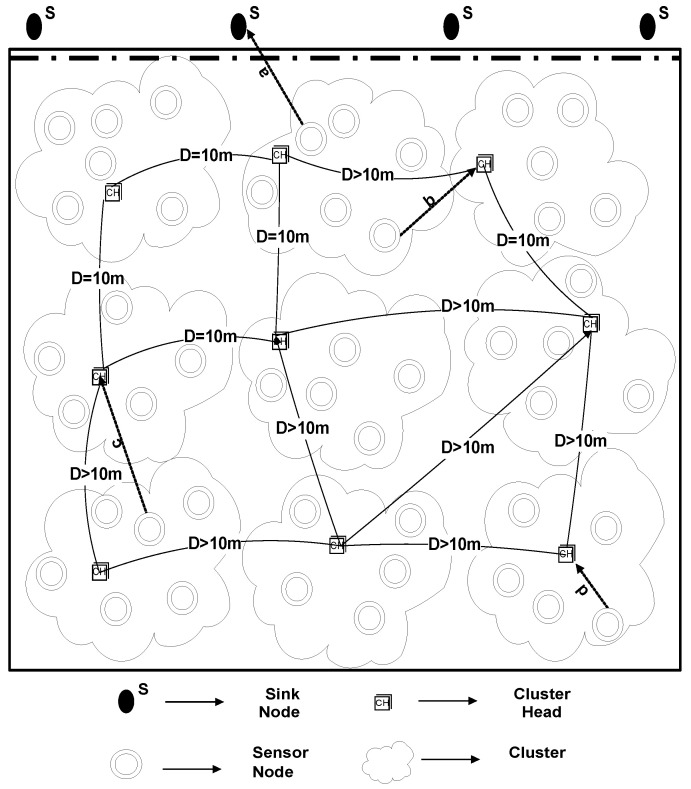
U-(ACH)${}^{2}$ with Four Sinks at Water Surface.

In our simulations, N = 225 sensor nodes are arbitrarily placed in a 500 m × 500 m × 500 m 3-D region and 4 sinks are positioned at the top of the water surface as shown in Figure 8. The sensor node and selected CH will send data to the minimum distance sink. The results are obtained from several runs in order to analyze the diversity among results. For each simulation, a very minute variation was found in the results, which were approximately equal to the previous simulation results. The reason behind the variation is the random deployment of sensor nodes. On average, the results were the same for each run. As the dimensions are 500 × 500 × 500, which is a small region for which 225 nodes are enough to work with. If the considered dimension was 1500 × 1500 × 1500, then we may have changed the number of nodes from 150 to 500 to examine the energy consumption of the overall network. We have considered 1000 rounds and, for each round, these nodes perform their desired activity. In the future, we will consider the expanded network region in order to observe the stability period of a network.

**Figure 9:**
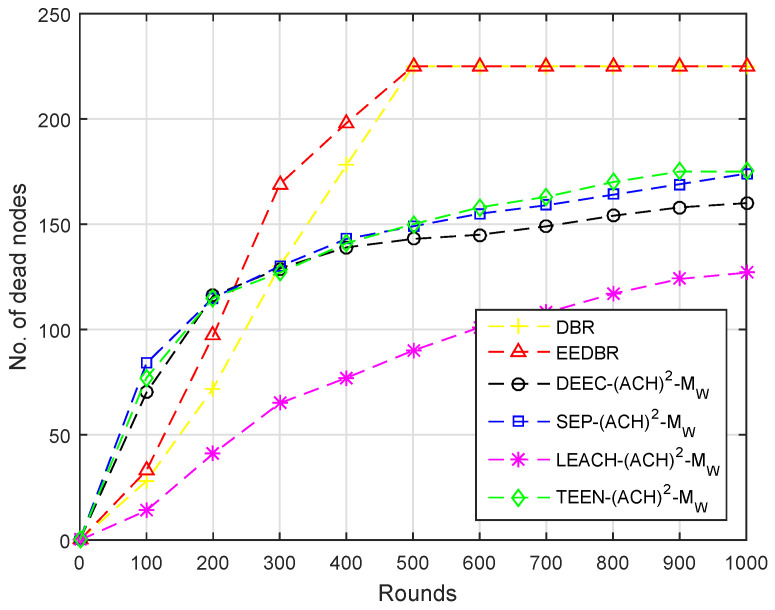
Number of Dead Nodes.

In Figure 9, almost all of the nodes of DBR and EEDBR died due to multiple forwarding. DEEC-(ACH)${}^{2}$, SEP-(ACH)${}^{2}$, TEEN-(ACH)${}^{2}$, and LEACH-(ACH)${}^{2}$ perform well in unstability period. In LEACH-(ACH)${}^{2}$, energy dissipation is uniform, hence a minimum number of nodes die in network lifetime.

**Figure 10:**
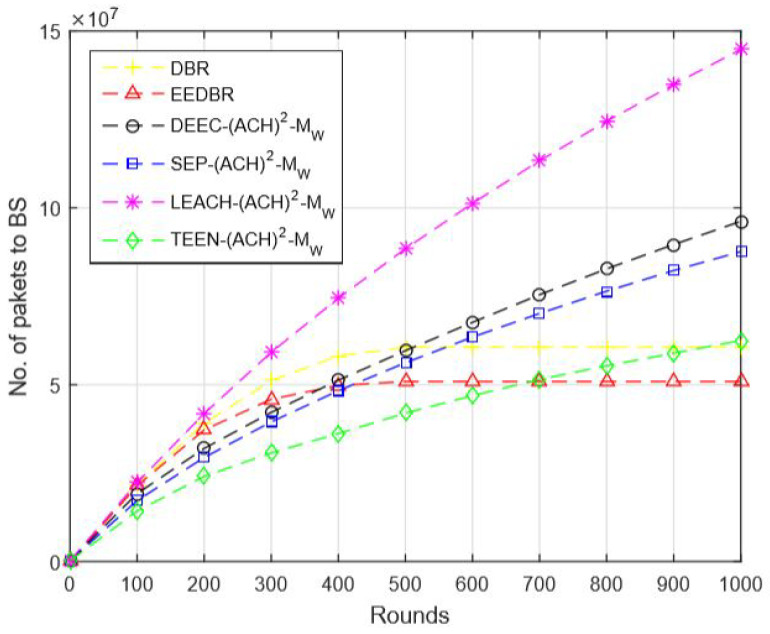
Number of Packets Sent to BS.

In Figure 10, DBR and EEDBR nodes send data to BS hop by hop. After a few rounds, nodes start dying, and few nodes cannot find a neighbor to forward data, which results in a limited number of packets conveyed to BS. LEACH-(ACH)${}^{2}$ achieves the best results in this network model.

**Figure 11:**
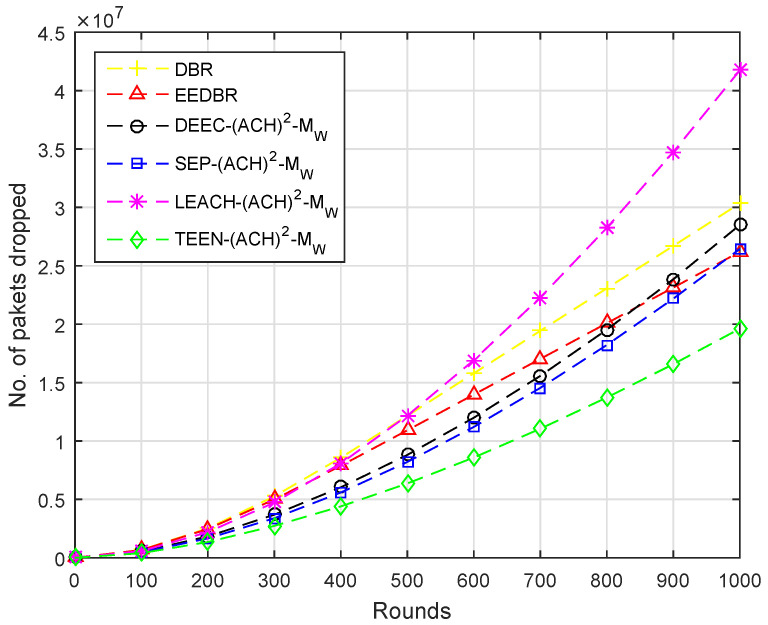
Number of Packets Dropped.

By comparing with other protocols, LEACH-(ACH)${}^{2}$ sends maximum packets to sink, hence, the packet dropped ratio is higher. In case of TEEN-(ACH)${}^{2}$, packets are sent to BS according to the threshold value, fewer packets are forwarded to BS; hence, less number of packets are dropped, as shown in Figure 11.

**Figure 12:**
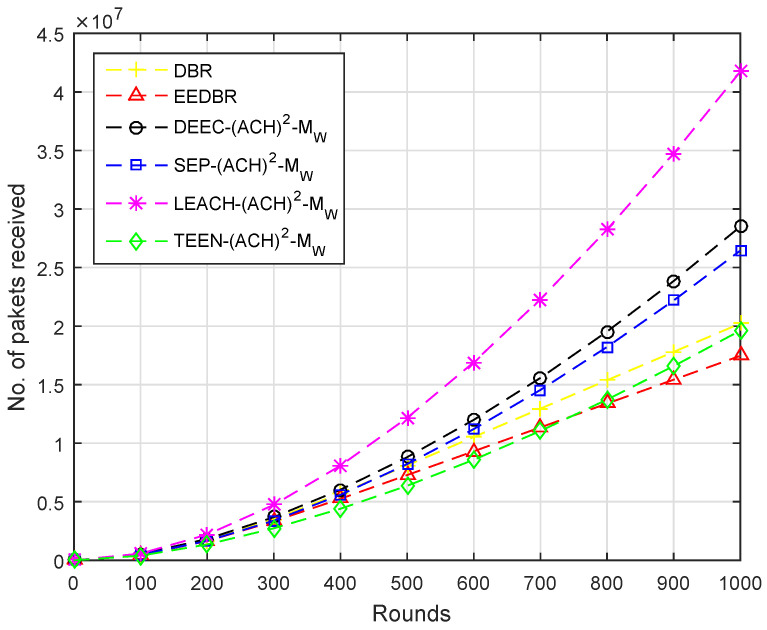
Number of Packets Received at BS.

In Figure 12, the number of packets received by each protocol are in the following order, LEACH$-{\left(ACH\right)}^{2}>$ DEEC-${\left(ACH\right)}^{2}>$ SEP$-{\left(ACH\right)}^{2}>$ DBR > TEEN$-{\left(ACH\right)}^{2}>$ EEDBR.

The performance of proposed schemes is demonstrated in Table 4 for scenario-II. It can be seen that the LEACH-(ACH)${}^{2}$ routing protocol outperforms here.

#### 4.1.3. U-(ACH)${}^{2}$ with One Sink at Water Surface and One Underwater

**Figure 13:**
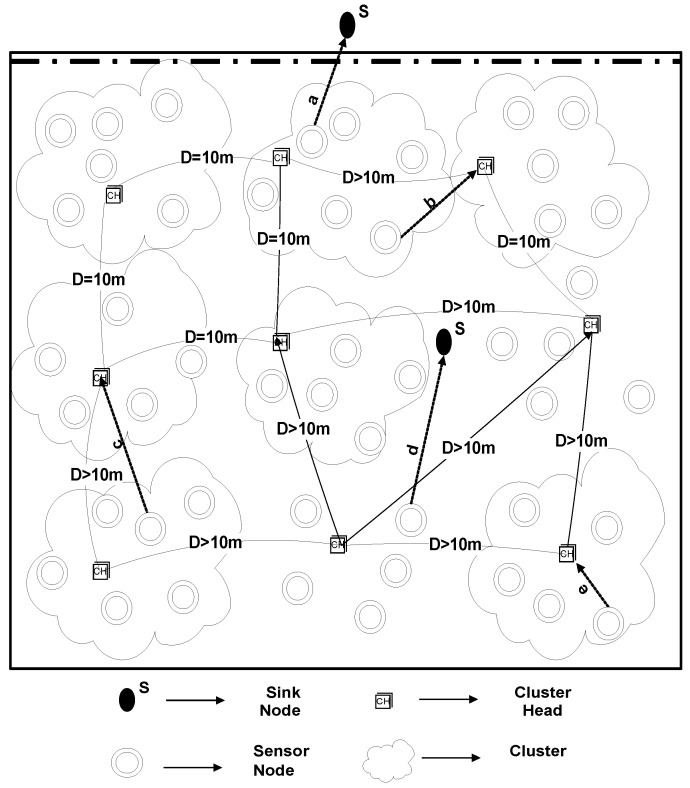
U-(ACH)${}^{2}$ with One Sink at Water Surface and One Underwater.

In our simulations, N = 225 sensor nodes are arbitrarily placed in a 500 m × 500 m × 500 m 3-D region, one of the sinks is placed in the middle of the water surface, and one is at top of the network. Sensor nodes having a depth greater than selected CHs and selected CHs will forward data to the nearer sink. CHs that are in the bottom of the network will forward data towards the sink, which is present in the middle of the network, as demonstrated in Figure 13. Likewise, previous two scenarios, here also the results were obtained from several runs to analyze the diversity among results. For each simulation, a very minute variation was found in the results which were approximately equal to the previous simulation results. The reason behind the variation is the random deployment of sensor nodes. On average, the results were the same for each run.

**Figure 14:**
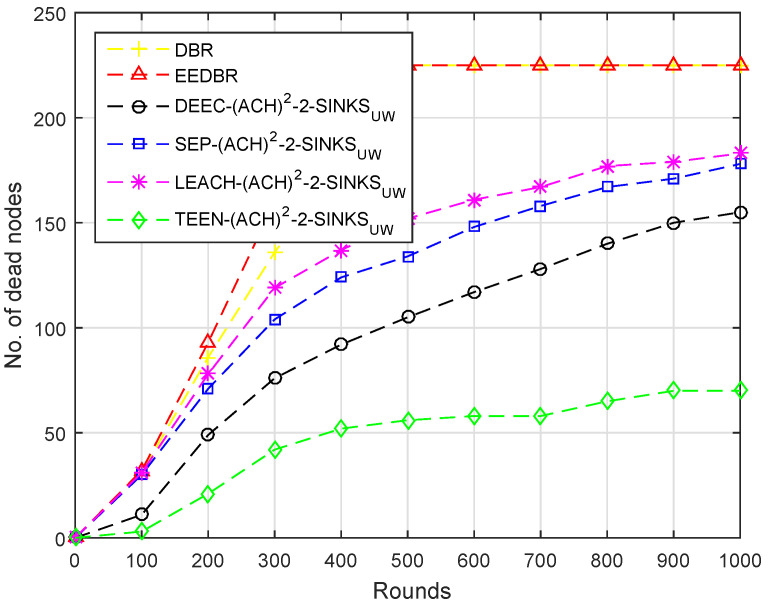
Number of Dead Nodes.

Due to multi-hop data forwarding, a maximum several nodes died in DBR and EEDBR, as shown in Figure 14. SEP-${\left(ACH\right)}^{2}$ and DEEC-${\left(ACH\right)}^{2}$ are heterogeneous routing protocols, thus nodes remain alive for a longer period. In TEEN-${\left(ACH\right)}^{2}$, less number of nodes die because nodes transmit data based on a threshold value.

**Figure 15:**
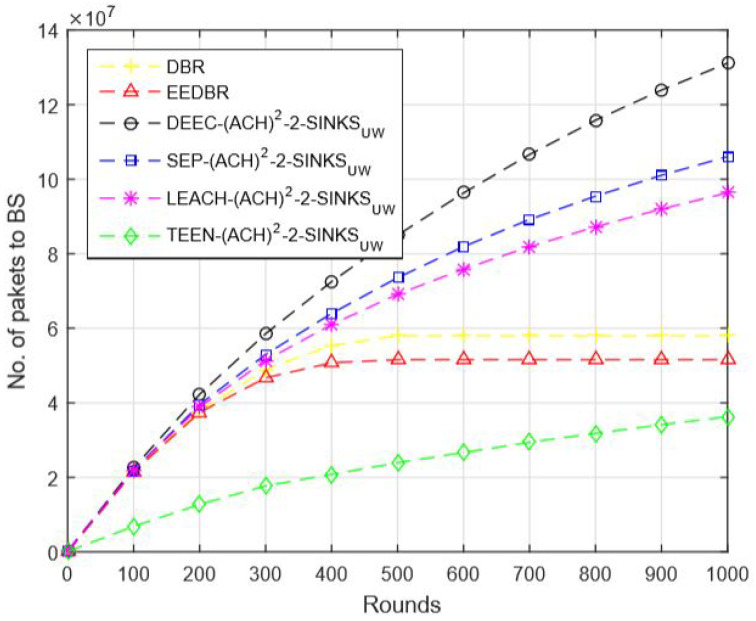
Number of Packets Sent to BS.

In Figure 15, data packets sent to sink are in order DEEC-${\left(ACH\right)}^{2}>$ SEP-${\left(ACH\right)}^{2}>$ TEEN-${\left(ACH\right)}^{2}>$ DBR > EEDBR > LEACH-${\left(ACH\right)}^{2}$, which is directly proportional to the remaining energy of a network.

**Figure 16:**
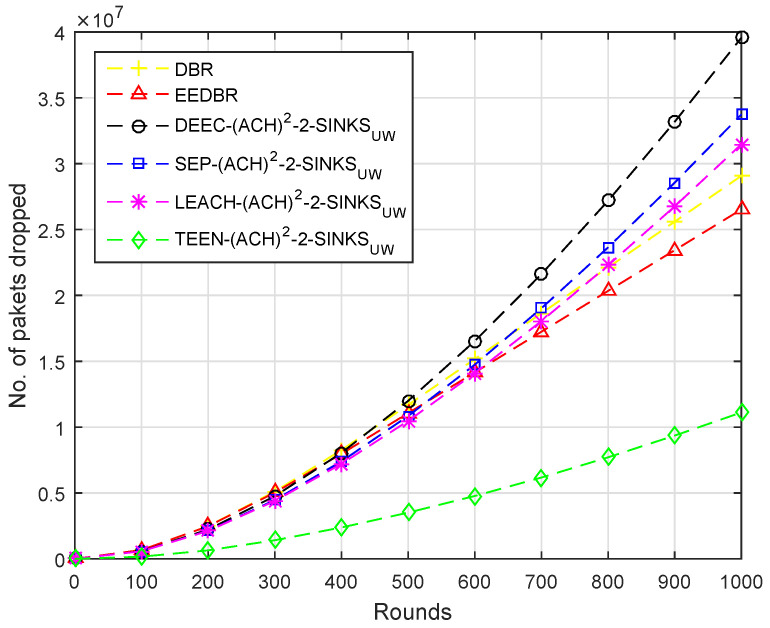
Number of Packets Dropped.

Figure 16 shows that the packet dropped is directly proportional to the packet sent to BS. In this case, the TEEN-${\left(ACH\right)}^{2}$ protocols perform much better than all other mentioned protocols.

**Figure 17:**
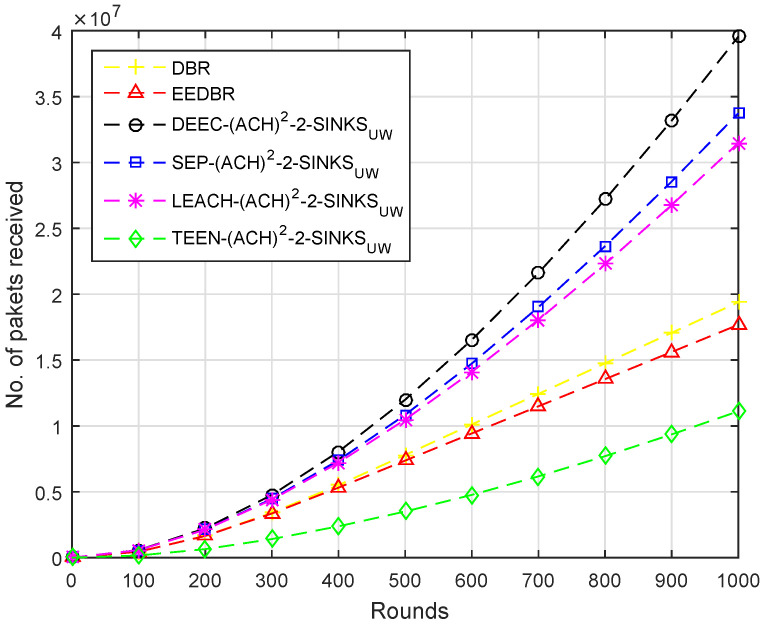
Number of Packets Received at BS.

According to Figure 17, packets received at the sink are in order DEEC$-{\left(ACH\right)}^{2}>$ SEP$-{\left(ACH\right)}^{2}>$ LEACH$-{\left(ACH\right)}^{2}>$ others. Less energy is consumed to transmit the packet to sink, as two sinks are deployed at top and underwater. The nodes having a depth greater than underwater sink will send data to it while others send data to the top sink.

The performance of proposed schemes is demonstrated in Table 5 for scenario-III. It can be seen that, concerning other routing protocols, DEEC-(ACH)${}^{2}$ routing protocol is still showing better performance than others.

In order to conduct the simulation, for each scenario, we have considered 1000 rounds and for each round, we have assumed the number of nodes ‘N’ as 225, as shown in the proposed study. We have considered 1000 rounds and for each round, these nodes perform their desired activity.

According to the results section, Table 6 is drawn, which shows the comparison of the number of discussed protocols. Any organization concerning routing protocols for transfer of data underwater can achieve their desired performance by considering any of the mentioned cases, i.e., deployment of sink nodes. Underwater routing protocols are performing varyingly in the considered cases. With respect to the existing protocols, the proposed protocols perform better. The trade-off is reflected in the performance of every considered protocol. However, according to the study, the DEEC-(ACH)${}^{2}$ protocol performs outclass among the number of mentioned protocols and, in the case where only one sink node is placed underwater and the other is implanted at the water surface.

In the proposed study, the challenge we face in each scenario is the harsh and unpredictable behavior underwater. Underwater, acoustic signals can cover more distance as compared to radio waves due to which acoustic signals are preferred. However, the scattering nature of water may affect the efficiency of our network.

With respect to the benchmarks, the proposed schemes perform better in almost all the considered scenarios. The purpose of considering 3 different scenarios is to get well informed before the implementation of a scheme. The installation of a scheme can be implemented in any of the three scenarios or maybe the organization has some constraints and wants to deploy sink nodes in a particular fashion then with the help of this study, the organization is able to find the optimal solution for its operation and so on. As it can be seen that for every scenario, the routing protocol performs differently.

In scenario-1, the DEEC-(ACH)${}^{2}$ protocol outperforms for most of the metrics of evaluation and generates promising results. DEEC-(ACH)${}^{2}$ shows the maximum number of packets are received at BS and less number of nodes died with respect to other routing protocols. However, a trade-off occurs between the maximum number of packets sent to the BS and packet drop ratio. DEEC-(ACH)${}^{2}$ is able to send the maximum number of packets to the BS due to which packet drop ratio is also high. In scenario-2, LEACH-(ACH)${}^{2}$ protocol performs better than all other schemes. Likewise, DEEC-(ACH)${}^{2}$ in scenario-1, here LEACH-(ACH)${}^{2}$ protocol acts similarly. LEACH-(ACH)${}^{2}$ shows the maximum number of packets received at BS and fewer nodes died in scenario-2. However, a tradeoff occurs between the maximum number of packets sent to the BS and packet drop ratio. LEACH-(ACH)${}^{2}$ is able to send the maximum number of packets to the BS due to which packet drop ratio is also high. In scenario.3, DEEC-(ACH)${}^{2}$ is able to send the maximum number of packets to BS, and the maximum number of packets are received at BS. For packet drop ratio TEEN-(ACH)${}^{2}$ performs best while less number of nodes died using SEP-(ACH)${}^{2}$. From these results, we have concluded that our proposed schemes have generated promising results and perform better than the benchmarks in each scenario.

## 5. Conclusions

In this study, the number of most recently used smart underwater routing protocols are analyzed. The terrestrial (ACH)${}^{2}$ routing protocol is integrated with a smart underwater routing scheme. Due to significant advancement in smart underwater development, the sensor network has gained a lot of researchers’ interest, but still, there are various situations where an optimal solution is required to be explored. The results of underwater implementation of (ACH)${}^{2}$ varies according to the acoustic modem type. In the proposed scheme, sink nodes are deployed in three different ways. In the first case, the only single sink is placed at the water surface. In the second case, four sinks are arranged and positioned at an equal distance from one another at the water surface. While, in the third case, one of the sinks is placed at the water surface and the second sink is deployed inside the predefined 3−D region. Moreover, all of the aforementioned cases are evaluated separately. Afterwards, the simulation results show that the maximum number of packets are favorably reached at the BS and less number of nodes died as compared to DBR and EEDBR. DEEC-(ACH)${}^{2}$ performs best generally, while, in case 2, LEACH-(ACH)${}^{2}$ performs best.

In the future, we intend to enhance our proposed scheme by considering link status and signal to noise ratio before sending any packets to BS. We are also interested in using the mobile sinks in our future study to support smart cities’ vision.

## Figures and Tables

**Table 1:** Comparison of Existing Protocols.

Protocol	Forwarder Node Selection Dependency	Routing	Energy Dependency	Operational Mode	Sink Mobility
DBR	Depth	Multi-hop	Homogeneous	Proactive	Static sink
EEDBR	Depth & residual energy	Multi-hop	Homogeneous	Proactive	Static sink
CDBR/CEEDBR	Depth	Multi-hop	Homogeneous	Proactive	Static sink
CoDBR	Depth	Cooperative	Homogeneous	Proactive	Static sink
RE-PBR	Depth & residual energy	Multi-hop	Homogeneous	Proactive	Static sink
iAMCTD	Depth, residual energy & SNR	Multi-hop	Homogeneous	Reactive	Static sink
DEADS	Depth & residual energy	Cooperative	Homogeneous	Reactive	Mobile sinks
DNAR & Co-DNAR	Depth & residual energy	Non-Coop & Cooperative	Heterogeneous	Proactive	Mobile sinks
EERD & Co-EERD	Depth & residual energy	Non-Coop & Cooperative	Heterogeneous	Proactive	Mobile sinks
LEACH	Residual energy	Clustering	Homogeneous	Proactive	Static sink
TEEN	Residual energy	Clustering	Homogeneous	Reactive	Static sink
SEP	Residual energy	Clustering	Heterogeneous	Proactive	Static sink
DEEC	Residual energy	Clustering	Heterogeneous	Proactive	Static sink

**Table 2:** Simulation parameters.

Parameters	Values
Network Region	500 m × 500 m × 500 m
Number of Nodes	225
Number of Sinks	1, 4, 2
Initial Energy	5 joule
Data Rate	10 kbps
Number of Rounds	1000

**Table 3:** Scenario-I: Performance of proposed schemes.

	TEEN-(ACH)${}^{2}$	DEEC-(ACH)${}^{2}$	SEP-(ACH)${}^{2}$	LEACH-(ACH)${}^{2}$
No. of dead nodes	high	less	high	high
No. of packets sent to BS	min	max	min	min
No. of packets dropped	less	high	high	high
No. of packets received	min	max	min	min

**Table 4:** Scenario-II: Performance of proposed schemes.

	TEEN-(ACH)${}^{2}$	DEEC-(ACH)${}^{2}$	SEP-(ACH)${}^{2}$	LEACH-(ACH)${}^{2}$
No. of dead nodes	high	high	high	less
No. of packets sent to BS	min	min	min	max
No. of packets dropped	less	high	high	high
No. of packets received	min	min	min	max

**Table 5:** Scenario-III: Performance of proposed schemes.

	TEEN-(ACH)${}^{2}$	DEEC-(ACH)${}^{2}$	SEP-(ACH)${}^{2}$	LEACH-(ACH)${}^{2}$
No. of dead nodes	high	high	less	high
No. of packets sent to BS	min	max	min	min
No. of packets dropped	less	high	high	high
No. of packets received	min	max	min	min

**Table 6:** Comparison of Proposed and Existing Protocols.

Protocol	Advances Achieved	Price to Pay	Working Principle
DBR	Minimum end to end delay, maximum throughput	Maximum energy consumption	Multi-hop
EEDBR	Minimum end to end delay, maximum throughput	Maximum energy consumption	Multi-hop
LEACH-${\left(ACH\right)}^{2}$	Average network lifetime	Throughput & max. energy consumption	Away clustering
DEEC-${\left(ACH\right)}^{2}$	Enhanced network lifetime, maximum packets send and received, less no. of dead nodes	Costly	Away clustering
TEEN-${\left(ACH\right)}^{2}$	Extends network lifetime	Throughput	Away clustering
SEP-${\left(ACH\right)}^{2}$	Extends network lifetime	Costly	Away clustering
